# Peracetic acid sterilized tendon and ligament allografts for knee reconstruction: For anterior cruciate ligament (ACL), posterior cruciate ligament (PCL) and complex knee surgery

**DOI:** 10.1007/s00132-024-04486-7

**Published:** 2024-03-18

**Authors:** Philipp Ahrens, Gudrun H. Borchert, Christin Freutel, Norus Ahmed, Jan C. Brune

**Affiliations:** 1 Orthoplus Munich, Alte Börse, Lehnbachplatz 2a, 80333 Munich, Germany; 2 Dr. Borchert Medical Information Management, Egelsbacher Str. 39e, 63225 Langen, Germany; 3 R&D, German Institute for Cell and Tissue Replacement (DIZG, gGmbH), Haus 42, Köpenicker Str. 325, 12555 Berlin, Germany

**Keywords:** Tendon, Allograft, ACL and PCL reconstruction, Larson, MPFL, Peracetic-acid, Sehnen und Bänder, Allograft, Rekonstruktion des vorderen und hinteren Kreuzbandes, Larson und MPFL, Peressigsäure

## Abstract

**Background:**

The use of allografts and autografts has been met with mixed views on whether allografts are a suitable alternative to autografts.

**Question:**

We aimed to investigate if chemically sterilized allografts show similar rerupture rates to those reported in the literature for allografts and autografts in anterior (ACL) and posterior cruciate ligaments (PCL) and complex knee surgery.

**Materials and methods:**

Retrospective data on knee reconstructions performed between 2011 and 2015 with tendon/ligamnet allografts sterilized with peracetic acid were collected in the form of a questionnaire. The inclusion criteria of 2 years for each patient were met by 38 patients, representing 22 ACL reconstructions, 5 PCL reconstructions, 3 OTHER surgeries, including the Larson technique and medial patellofemoral ligament (MPFL) reconstruction and 8 COMPLEX surgeries. The main endpoints were rerupture and complication rate. Secondary endpoints included stability of the knee (Lachman test, Pivot shift test) and the range of motion.

**Results:**

The rerupture rate was 7.9% (3 grafts). Reruptures only occurred in the ACL group. No reruptures were observed in the PCL, OTHER and COMPLEX surgery groups. Stability improved significantly after surgery and the range of motion returned to values similar to that of healthy knees.

**Conclusions:**

Tendon allografts sterilized with peracetic acid show promising low rerupture rates and good clinical scores and the results are comparable to the literature on autografts and other allografts.

**Graphic abstract:**

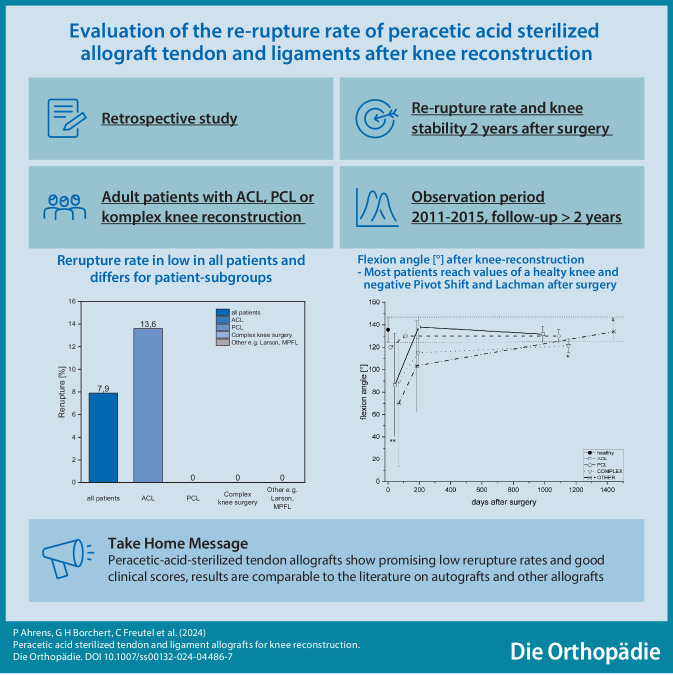

## Zuammenfassung

**Hintergrund:**

Bei der Verwendung von Allotransplantaten und Autotransplantaten gibt es unterschiedliche Meinungen darüber, ob Allotransplantate eine geeignete Alternative zu Autotransplantaten sind.

**Fragestellung:**

Weisen chemisch sterilisierte Allografts bei der Rekonstruktion des vorderen (VKB) und/oder hinteren (HKB) Kreuzband und komplexen Knieoperationen ähnliche Rerupturraten auf wie die in der Literatur angegebenen Werte für Allografts und Autografts?

**Material und Methoden:**

Daten zu Knierekonstruktionen, die zwischen 2011 und 2015 mit Peressigsäure-sterilisierten Sehnenallotransplantaten durchgeführt wurden, wurden in Form eines Fragebogens gesammelt. Die Einschlusskriterien von 2 Jahren für jeden Patienten wurden von 38 Patienten erfüllt, die 22 VKB-Rekonstruktionen, 5 HKB-Rekonstruktionen, 3 andere Operationen, einschließlich Larson-Technik und Medial Patello-Femoral Ligament (MPFL)-Rekonstruktion, und 8 komplexe Operationen repräsentierten. Die wichtigsten Endpunkte waren die Reruptur- und Komplikationsraten. Zu den sekundären Endpunkten gehörten die Kniestabilität (Lachman-Test, Pivot-shift-Test) und der Bewegungsumfang nach der Neutral-0-Methode.

**Ergebnisse:**

Die Rerupturrate betrug 7,9 % (3 Transplantate). Rerupturen traten nur in der VKB-Gruppe auf. In den Gruppen HKB, OTHER und COMPLEX wurden keine Rerupturen beobachtet. Die Stabilität verbesserte sich nach der Operation deutlich, und der Bewegungsumfang erreichte wieder Werte, die denen gesunder Knie ähnlich waren.

**Schlussfolgerung:**

Mit Peressigsäure sterilisierte Sehnen-Allotransplantate zeigen vielversprechend niedrige Rerupturraten und gute klinische Ergebnisse, die mit der Literatur über Autotransplantate und andere Allotransplantate vergleichbar sind.

## Introduction

Autografts are the graft of choice for primary reconstruction of the anterior cruciate ligament (ACL); however, the availability of autografts is limited, and sizes may be unpredictable or insufficient depending on the surgical requirements. Over the years, allografts have provided a safe alternative due to improved processing methods and an abundant supply of grafts. The advantages of such grafts include attenuated donor site morbidity [13], shorter duration of surgery [13, 18, 22], smaller incisions, and a wider selection of graft sizes [4]. Some disadvantages may include slower biological remodelling and a theoretical risk of disease transmission [4] that can be minimized by using chemically sterilized allografts. Both allografts and autografts have reported similar functional outcomes in 10-year follow-up studies [15] and in reports with shorter follow-up [1, 6–8, 10, 16–19]. Allograft use is well reported for both anterior (ACL) [1, 2, 9, 10, 15, 19] and posterior cruciate ligament (PCL) reconstruction [16].

The aim of this study is to investigate whether tendons sterilized with peracetic acid used in knee surgery display similar rerupture and complication rates to values reported in the literature for other allografts and autografts.

## Material and methods

### Participants

For 38 patients, representing 22 ACL reconstructions, 5 PCL reconstructions, 3 other surgeries, including Larson and medial patellofemoral ligament (MPFL) reconstructions and 8 complex surgeries we could collect data with a follow-up of at least 2 years. After-treatment of the patient is mainly not performed by the operating surgeon. Thus, the data after 2 years were not available for many patients treated with the tendon/ligament allograft. The mean age was between 31–39 years and the age range was between 14–55 years, depending on the surgery group. The mean body mass index (BMI) for each group was between 27 and 31 kg/m^2^, and the range was between 20–37 kg/m^2^ (Table 1). A total of 21% of the patients were female and the left knee was involved in 39% of the patients. For 29 patients (76%) a traumatic rupture was recorded, for 3 patients the surgery was performed due to remaining instability from previous ACL reconstructions/osteotomies and for the remaining 6 patients (16%) the data were not available. In patients where traumatic injury was reported, sport was the reason for 50% of the cases. Comorbidities were noted for 7 patients (Table 1). Due to the low number of reruptures a gender-based investigation was not performed.

**Table 1:** Presurgical patient demographics

**Patient data**	**Mean** **±** **SD (*n*)**
*Age, years*	
ACL	33.7 ± 10.4 (22)
PCL	39.4 ± 3.9 (5)
COMPLEX^a^	30.8 ± 14.2 (8)
OTHER^b^	32.0 ± 9.8 (3)
*BMI, kg/m*^*2*^	
ACL	27.2 ± 4.1 (18)
PCL	27.8 ± 2.9 (5)
COMPLEX	30.9 ± 5.5 (6)
OTHER	26.6 ± 9.2 (3)
**Patient data**	**Number (%)**
*Gender*	
Male	30
Female (%)	8 (21)
*Left knee/right knee*	
ACL	9/13
PCL	2/3
COMPLEX	3/5
OTHER	1/2
*Traumatic rupture (yes/no/no data)*	
ACL	17/2/3
PCL	5/0/0
COMPLEX	6/0/2
OTHER	1/1/1
*Injured during*	
(sport/traffic/work/home/others/no data)	14/4/7/1/2/10
Smoker (yes/no/no data)	7/17/14
Comorbidities	7

^a^*COMPLEX* complex surgeries include: ACL + PCL, PCL + collateral ligaments

^b^*OTHER* other surgeries include: Larson plastic surgery, medial ligament plastic, MPFL

### Grafts

All allografts analyzed were provided by the German Institute for Cell and Tissue Replacement (DIZG). They are sterilized using a validated, GMP-compliant process and are approved as medicinal products under § 21 of the German Medicinal Products Act (PEI.H.03356.01.1). All tissues are acquired from non-profit tissue recovery partners after providing informed consent. Tendons and ligaments from a single donor are thawed at 2–8 °C and remnants of blood, fat and connective tissue are removed. For sterilization, tissues are fully submerged in validated tissue-preserving sterilization solution (2% peracetic acid, 96% ethanol, aqua-dest; ratio v/v/v|2/1/1) and incubated with constant agitation, at low pressure and at room temperature for 4 h. Subsequently, tissues are rinsed in a washing process using aqua-ad-iniectabilia. Under aseptic conditions (class A clean rooms) the sterile grafts are then transferred into primary and secondary packaging. The allografts are stored at −40 °C and can be used for up to 2 years.

### Questionnaire design

A questionnaire was developed and sent to physicians who had used tendon allografts sterilized with peracetic acid for knee surgery in Germany. This questionnaire had to be completed in a pseudonymized way. Only the surgeon completing the questionnaire is able to identify the patient. The biometrician cannot identify the patient because on the questionnaire there is no date of birth and no date of surgery. Because this questionnaire was sent from the manufacturer of the graft, it was not the scope of the investigation to ask for results with grafts from competitors/autologous grafts. For this reason, there was no control group and only results described in the literature were used for comparison. The main focus of the study was to show that this kind of allograft can be used for the reconstruction of the knee. The first part of the questionnaire included patient data and the surgical procedure, and the second part included data obtained during follow-up, such as clinical outcomes (Lachman test and Pivot shift test for ACL patients and ROM), possible reruptures (reruptures were defined when Lachman or Pivot shift tests were ≥ 2 or when MRI confirmed the rerupture) and any complications recorded during the follow-up time. A total of 38 grafts including 22 ACL reconstructions, 5 PCL reconstructions, 8 COMPLEX surgeries (ACL + PCL, PCL + collateral ligaments) and 3 grafts for OTHER surgeries (Larson technique, medial ligament plastic and MPFL reconstruction) met the inclusion criteria.

### Data collection

The study was exempt from institutional review board approval by the Bayrische Landesärztekammer under the sign 2022-1153 as direct involvement of the patients was not required. All data were collected anonymously. Due to the retrospective nature of this analysis some data were not available for all patients. For each parameter, the numbers included are given in the tables. The inclusion criteria are defined as the use of a peracetic-acid-sterilized allograft, an operation time between 2011–2015 and at least 2 years of follow up. The exclusion criteria included non-peracetic-acid-sterilized allografts, operation dates not between 2011–2015 and less than 2 years follow up.

### Calculations and statistics

Values are given as mean ± SD, with range, or median values, calculated with Prism for MacOSX (version 7.0e, GraphPad Software Inc., San Diego, CA, USA). Continuous values were analyzed with a nonparametric Mann-Whitney tests for 2‑group comparisons, because of non-Gaussian distribution of the data. For ordinal or categorial values, contingency tables were used, and odds ratios were calculated. Kruskal-Wallis tests of continuous values were performed for comparison of 3 or more groups combined with Dunn’s multiple comparisons test. A *p*-value < 0.05 was defined as significant and levels of significance are indicated as follows: **p < 0.01, *p < 0.05.

## Results

Mean time to surgery was 10–83 months, depending on the group investigated (Table 2). Primary surgery was performed in 34% of the patients. The remaining 66% were revisions. The mean duration of surgery was 89–142 min (Table 2). Patients who underwent complex surgery stayed significantly (*p* = 0.01658) longer in the hospital than patients with ACL reconstruction.

**Table 2:** Presurgical clinical data

Clinical data	Mean ± SD (*n*)
*Time to surgery, months*	
ACL	15 ± 33 (19)
PCL	83 ± 92 (5)
COMPLEX^a^	41 ± 88 (7)
OTHER^b^	10 ± 4 (2)
*Primary surgery, n, (%)*	
ACL	7 (32)
PCL	1 (20)
COMPLEX	3 (38)
OTHER	2 (67)
*ASA classification*	
(I/II/III/IV/V/VI/no data)	19/10/1/0/0/0/8
*Associated injuries (yes/no/no data)*	
ACL	12/6/4
PCL	2/1/2
COMPLEX	7/0/1
OTHER	1/0/2
*Duration of surgery, min*	
ACL	109.8 ± 41.1 (12)
PCL	92.6 ± 24.9 (5)
COMPLEX	142.0 ± 62.4 (6)
OTHER	88.7 ± 30.9 (3)
*Hospital stay, days*	
ACL	2.9 ± 1.6 (22)
PCL	4.4 ± 0.9 (5)
COMPLEX	10.9 ± 8.1 (8)*
OTHER	5.0 ± 1.0 (3)
	*p = 0.01658 COMPLEX vs. ACL

^a^*COMPLEX* complex surgeries include: ACL + PCL, PCL + collateral ligaments

^b^*OTHER* other surgeries include: Larson plastic, medial ligament plastic, MPFL

Follow-up was at least 2 years for each surgical treatment and mean time was between 48 and 66 months and not significant different between groups (Table 3). The complication rate was 7.9%, none of these complications were related to the allograft (Table 3).

**Table 3:** Follow-up and postsurgery data

Patient data	Mean ± SD(*n*)	
*Follow-up, months*		
ACL	66 ± 20 (22)	
PCL	48 ± 25 (5)	
COMPLEX^a^	58 ± 28 (8)	
OTHER^b^	50 ± 25 (3)	
**Patient data**	**Number (%)**	
Complications	1 redness	
	2 hemarthrosis	
	(none of these associated with the graft)	
*Reruptures, rate n: (ruptured/all (%))*		
ALL	3/38 (7.9)	
ACL	3/22 (13.6)	
PCL	0/5 (0)	
COMPLEX	0/8 (0)	
OTHER	0/3 (0)	
*Lachman (ACL)*	*Presurgery*	*At last follow-up*
0	1	15
1	3	2
2	15	0
3	1	0
*Pivot shift (ACL)*	*Presurgery*	*At last follow-up*
0	0	10
1	13	0
2	1	0
*Flexion angle (Range of motion)* at last follow-up		
Healthy knee	135.6°± 10.9°	
ACL	131.7°± 7.2°	
PCL	130.0°± 6.1°	
COMPLEX	121.2°± 6.4°**	
OTHER	133.9°± 7.0°*	
	**p = 0.00072 COMPLEX vs. healthy knee	
	*p = 0.04553 COMPLEX vs. OTHER	
*Mean time to rerupture*		
ACL	54 ± 7 months (3)	
*Rerupture in primary surgery (n/%)*		
ACL	1 (33%)	
*Retrauma (yes/no)*		
ACL	1/2	
**Patient data**	**Mean** **±** **SD (*n*)**	
*Donor age, years (ACL)*		
Nonruptured	54.1 ± 6.9 (19)	
Ruptured	55.6 ± 4.6 (3)	
	*p* = 0.84804	
*Patient age, years (ACL)*		
No reruptures	33.8 ± 10.9 (19)	
Reruptures	32.7 ± 7.5 (3)	
	*p* = 0.70058	
*Patient BMI, kg/m*^*2*^* (ACL)*		
No reruptures	26.4 ± 4.0 (19)	
Reruptures	30.8 ± 2.0 (3)	
	*p* = 0.0659	

^a^*COMPLEX* complex surgeries include: ACL + PCL, PCL + collateral Ligaments

^b^*OTHER* other surgeries include: Larson plastic, medial ligament plastic, MPFL

We recorded 3 reruptures (7.9%), of which 2 grafts were reruptured in one patient, due to the noncompliance of a multiple revision patient. Reruptures only occurred in the ACL patient group.

In ACL patients, presurgical Lachman test was positive with 2+ or higher in 80% of the patients and pivot shift test was positive in 100% of the patients, when data were available (Table 3). Postsurgery all patients had a negative pivot shift test and a Lachman test of < 2+. Due to insufficient data, KOOS and IKDC could not be analyzed.

Donor age, patient age and BMI did not differ for patients with and without rerupture (*p* = 0.84804; *p* = 0.70058 and *p* = 0.0659, respectively, Table 3).

**Fig. 1:**
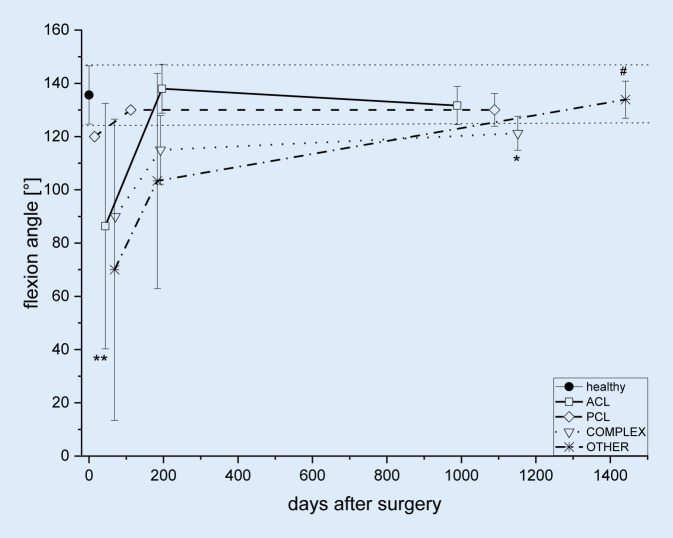
Flexion angle (ROM) of the knee for different surgical procedures: flexion angle [°] of the healthy knee (*closed symbol*) and treated knee groups (*open symbols*) over time. Data are presented as mean ± SD, **p < 0.00335 vs. healthy, *p < 0.04553 vs. healthy, #*p* < 0.00072 vs. COMPLEX. *ACL* open square, *PCL* open diamond, *COMPLEX surgery* open triangle, *OTHER procedures* star

Flexion angles (ROM) of 16 healthy knees were obtained from the contralateral side. These values were used as the reference. The ACL and PCL patients recorded flexion angles in the normal range with 138° and 130° of flexion, respectively, 6 months after reconstruction (Fig. 1). The COMPLEX and OTHER surgery groups showed lower flexion angles after 6 months due to longer restriction of movements for these patient groups (Fig. 1). At the final follow-up (Table 3, Fig. 1) patients in the ACL, PCL and OTHER surgery groups had similar values to the healthy knee with 132°, 130° and 134° flexion, respectively. Patients in the COMPLEX group showed a lower flexion angle (121°) compared to the healthy knee reference (*p* = 0.00072) and to the OTHER surgery group (*p* = 0.04553).

For the 38 patients the allografts used were 32 semitendinosus tendons, 1 gracilis tendon, 1 tibialis posterior tendon, 3 ligamentum patella with bone block and 1 ligamentum patella tendon, preshaped. The allograft tendons were used for a variety of procedures including, e.g., the reconstruction of the ACL, PCL, ACL + PCL, Larson plastic surgery and the medial patellofemoral ligament (MPFL). No correlation was found between rerupture and donor age (Table 3).

## Discussion

The most important finding of this retrospective data analysis is that allografts sterilized with peracetic acid can be used for different kinds of knee reconstructions with a success rate of 92%. A 100% survival rate of the allograft was recorded for PCL reconstruction, complex surgeries like simultaneous ACL + PCL, PCL and collateral ligaments, Larson plastic surgery, medial ligament plastic and MPFL reconstruction. The meta-analysis of Migliorini et al. [16] summarized that allografts can be considered a suitable alternative to autografts for PCL reconstruction. For ACL reconstruction the minimum follow-up was 32 months, survival of 86% after a median time of 66 months (5.5 years) was recorded with low complication rates not related to the allograft. Knees treated with allografts sterilized with peracetic acid showed good flexion angles with a negative Pivot shift test and Lachman test values below 2. We could confirm the findings of Ashton et al. [2] that there is no correlation between the donor age and rerupture rate. Allografts provide an acceptable alternative to autografts for reconstructive surgery [16]. Allografts aid in reducing graft harvest site morbidity and provide surgeons with an abundant supply of grafts [13], where the type of graft can be selected according to the patient requirements (diameter, length); however, the literature varies regarding rupture rates and on the use of allografts and autografts with no apparent differences between allografts or autograft use (1–12% for allograft and 1–13% for autograft [1, 10, 11, 18, 19, 26]) and some reports displaying higher rerupture rates for allografts (7–26% [6, 8]); however, in this retrospective analysis the use of chemically sterilized allografts displayed results similar to previous reports (6.6–13% for autografts and 6.5–12% for allografts, regardless of the sterilization method [7, 17, 18, 25]).

The mean follow-up (Table 3) was between 48–66 months and the range was between 24–95 months. All complications resolved and did not lead to ruptures. The mean time to failure of allografts used in this study was 54 months and is higher compared to other reports (18–22 months [23]), but lower than reported by Macchiarola et al. (6.8 ± 5.4 years [15]).

For most of the cases presented here (84%), semitendinosus tendon allografts were used. Semitendinosus graft use alone was only described in autografts [25] or in combination with gracilis grafts for allografts and autografts [9, 11, 20, 22, 24]. Interestingly, in this cohort semitendinosus tendons were also used for reconstruction of the MPFL or the collateral ligament. This displays the wide usability of chemically sterilized allografts; however, complications arise in both allografts and autografts and the complication rate in this cohort was 7.9% (not related to the allograft) with previous literature reporting rates ranging between 1.7–13% [11]. Previous biomechanical and other studies have supported that donor age is not correlated to the rerupture rate [2]. This follows our observations with no correlation between donor age and the rerupture rate.

This retrospective study also included secondary outcomes such as knee stability, range of motion (ROM) and did not focus on a specific group of patients such as athletes. The mean age of all patients was 34 years (range 14–55 years) and was within the range of published data, literature displaying lower age (27–33 years) [20]. The average BMI of all patients was 28 kg/m^2^ (range 20.1–37.2 kg/m^2^) and was higher than in other studies [14, 20]. This must be considered when evaluating rerupture rates as it correlates with the BMI. Physical activity (i.e., sports) was the main reason for injury (50%) similar to other reports [7, 8, 18]. Investigations on knee stability and ROM within this cohort displayed a reduction in Lachman grading postsurgery as shown in previous reports using both autografts and allografts [12]. The number of patients with Lachman grading 2 or more prior to surgery (80% of patients) and postsurgery (0%) was within the reported ranges for presurgery (43–100% [7, 9, 14, 18, 24, 26]) and postsurgery (0–22%) for allografts and autografts [7, 9, 14, 18, 20, 24, 26]. A long-term follow-up reported autografts to display a lower negative Lachman grading at 10 years postoperation (31% [17]).

Positive Pivot shift was recorded in all patients before surgery and in none of the patients after surgery, which follows previous reports [22] while several groups reported a positive pivot shift postsurgery (5–15% [11, 18, 21]).

The ACL reconstruction only cohort (Fig. 1) displayed a flexion angle (138° after 6 months) similar to the healthy knees as described in previous reports [25]. Flexion in the PCL group was 130° which was confirmed by Migliorini et al. [16] with 131.7°. Flexion in the COMPLEX and OTHER-surgery groups reached a normal range later due to movement restrictions in these groups after surgery. Interestingly, Bach et al. [3] reported a similar outcome of 140° 51 months postoperation while Barret et al. [5] reported an average flexion of 121.1° after 2 years. The data presented here on stability and ROM of the knee are well within the range when compared to previous reports for allografts and autografts [3, 5, 25] irrespective of the allograft used and the procedure performed. In the literature, both non-irradiated and chemically sterilized allografts showed similar results to autografts with respect to activity scores and stability [21].

### Limitations of the study

The retrospective nature of this data made it impossible in almost all cases to access complete data sets. Insufficient resources at the hospitals proved to be the main reason. Due to the inclusion criteria only 38 patients with a follow-up of more than 2 years could be delivered by the contacted surgeons. This is due to the shorter follow-up time for knee reconstruction in daily orthopedic practice. Patients with satisfactory results often omit seemingly unnecessary follow-ups. On the other hand, unsatisfied patients may change the attending physician. In both cases, patients are lost to follow-up. Furthermore, the lack of a control group does not allow a direct in-study comparison, thus referring to previous literature reports.

## Conclusion

Chemically sterilized allografts are a promising alternative to autografts for ACL, PCL reconstruction as well as complex knee surgery. Further studies have to be carried out to underline these findings. All evaluated data were in the range reported in the literature (for allografts) and do not differ greatly from reports on autografts.

## Abbreviations

ACL: anterior cruciate ligament
PCL: posterior cruciate ligament
MPFL: medial patellofemoral ligament
